# The recognition of ubiquitinated proteins by the proteasome

**DOI:** 10.1007/s00018-016-2255-5

**Published:** 2016-05-02

**Authors:** Guinevere L. Grice, James A. Nathan

**Affiliations:** Department of Medicine, Cambridge Institute for Medical Research, University of Cambridge, Cambridge, CB2 0XY UK

**Keywords:** Proteasome, Ubiquitin, Polyubiquitin chains, Ubiquitin binding protein, Ubiquitin binding domain, Ubiquitin receptors

## Abstract

The ability of ubiquitin to form up to eight different polyubiquitin chain linkages generates complexity within the ubiquitin proteasome system, and accounts for the diverse roles of ubiquitination within the cell. Understanding how each type of ubiquitin linkage is correctly interpreted by ubiquitin binding proteins provides important insights into the link between chain recognition and cellular fate. A major function of ubiquitination is to signal degradation of intracellular proteins by the 26S proteasome. Lysine-48 (K48) linked polyubiquitin chains are well established as the canonical signal for proteasomal degradation, but recent studies show a role for other ubiquitin linked chains in facilitating degradation by the 26S proteasome. Here, we review how different types of polyubiquitin linkage bind to ubiquitin receptors on the 26S proteasome, how they signal degradation and discuss the implications of ubiquitin chain linkage in regulating protein breakdown by the proteasome.

## Introduction

Diversity in the ubiquitin system is generated by the ability of ubiquitin to form eight different types of chains on itself through its seven lysine (K) residues (K6, K11, K27, K29, K33, K48, and K63) or N-terminus (Met-linked or linear). Further complexity is generated by the formation of polyubiquitin chains of uniform linkage (homotypic) or mixed linkages (heterotypic). Each type of polyubiquitin chain has the potential to act as a distinct intracellular signal that must be specifically identified and decoded by ubiquitin binding proteins (UBPs), to facilitate the diverse outcomes of ubiquitination within cells (reviewed in [1]). This is typically exemplified by the role of K48-polyubiquitin chains signalling proteasomal degradation, while K63-polyubiquitin conjugates are involved in non-proteasomal pathways, including intracellular signalling, DNA repair, and the endosomal–lysosomal system.

Degradation of intracellular soluble proteins is principally mediated by the 26S proteasome. This 2.5 MDa complex comprises the cylindrical 20S chamber, where proteolysis occurs, and the 19S regulatory particle, which binds polyubiquitinated proteins and governs their entry into the 20S catalytic chamber. Through its six ATPase subunits, the 19S complex catalyzes gate opening in the 20S outer ring, substrate unfolding, and translocation into the 20S’s internal proteolytic chamber. Polyubiquitinated substrates are recognized, tightly bound, and efficiently degraded by the eukaryotic 26S through several carefully integrated steps: (1) The initial binding step involves high affinity ubiquitin receptor sites [19S subunits Rpn10 (S5a) and Rpn13] [2–4]. This step is ATP-activated but is easily reversible [2]. (2) Some chains then become more tightly bound and committed to proteolysis, through a process requiring both an unfolded domain within the ubiquitinated protein and ATP hydrolysis [2]. (3) Proteins are deubiquitinated by one or more of the 26S-associated deubiquitinating enzymes (DUBs), Usp14, Uch37, or Rpn11 [5]. (4) Finally, the protein is unfolded and translocated through the opened gate of the 20S [6–8].

While K48-linked polyubiquitin chains are established as the canonical signal for proteasomal degradation, recent studies highlight the ability of the proteasome to bind monoubiquitinated proteins or other types of polyubiquitin chains. Here, we discuss how ubiquitinated proteins bind to the 26S proteasome, and focus on the ability of the proteasome to distinguish between different polyubiquitin linkages.

## Proteasome ubiquitin receptors

The outcome of polyubiquitination is dependent on recognition of the ubiquitin chains by UBPs, which act as ubiquitin receptors. These proteins usually encode several ubiquitin-binding domains (UBDs) that may differ in their affinity and avidity for distinct ubiquitin linkages. UBDs typically consist of one or more α-helices that bind ubiquitin through a hydrophobic region, centered on isoleucine-44 (I44). Most UBDs of the α-helical class have relatively low affinities for a single ubiquitin molecule, but micromolar affinities for tetraubiquitin chains [9]. The presence of several UBDs within the same protein not only increases the affinity for polyubiquitin chains but can also change the avidity and determines linkage selectivity (reviewed in [10]).

**Fig. 1:**
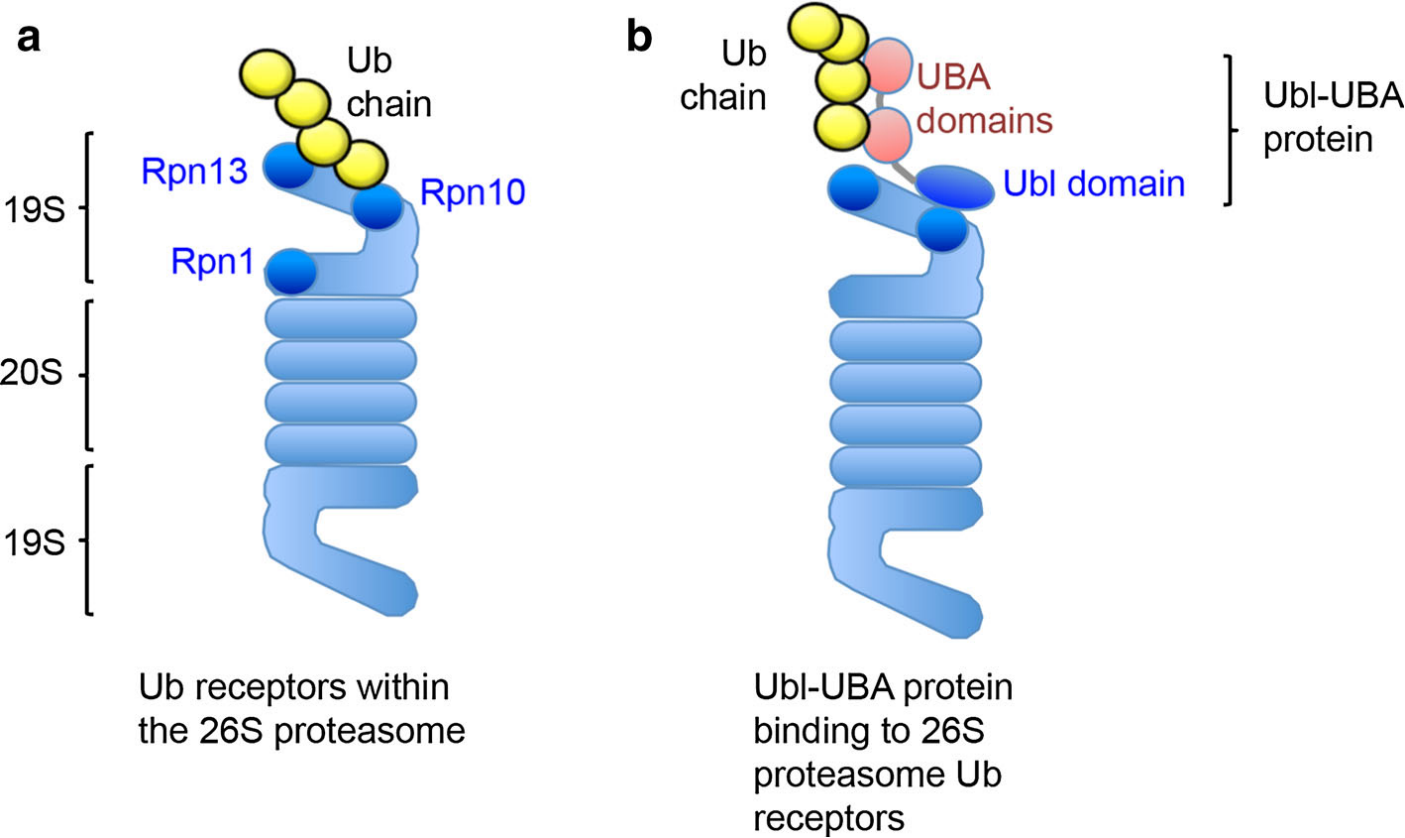
Proteasome ubiquitin receptors. Schematic of the 26S proteasome illustrating the position of the intrinsic proteasome receptors (**a**) and the association of the UBA–Ubl proteins with the 19S proteasome (**b**). Rpn10 and Rpn13 are the predominant high affinity sites for binding ubiquitinated proteins. In yeast, Rpn1 may also facilitate binding of ubiquitinated proteins to the 19S (**a**). Ubl–UBA proteins, such as Rad23 and Dsk2, associate with the 26S through their Ubl domains that bind to either Rpn10 or Rpn13. The UBA domains bind the polyubiquitin chains (**b**)

The first ubiquitin receptor identified within the 26S proteasome was Rpn10 (S5a). Deveraux et al. used proteasomes isolated from human red blood cells and observed that the 19S subunit Rpn10 bound ubiquitinated lysozyme conjugates [11]. However, this was clearly not the only ubiquitin receptor for the proteasome, as the deletion of Rpn10 in *saccharomyces cerevisiae* had a modest phenotype and did not affect viability [12]. Subsequent studies in yeast have been instrumental in identifying other UBDs within other intrinsic components of the proteasome, namely, Rpt5, Rpn13, and Sem1 (Dss1) [4, 13, 14] (Fig. 1a; Table 1). While all these subunits can bind polyubiquitinated proteins, the relative contribution of each for facilitating proteasomal degradation is unclear. However, biochemical assays of ubiquitin binding and protein degradation show that the 19S subunits, Rpn10 and Rpn13, are the high affinity ubiquitin receptors for the proteasome (Fig. 1a) [2–4, 15].

**Table 1:** Proteasome ubiquitin receptors

Gene	Species	Ubiquitin binding domains	Location within the 26S proteasome	Assigned functions
Rpn10	Yeast	Single ubiquitin-interacting motif (UIM) domain	Intrinsic 19S subunit, but can be substoichiometric	Binds K48 and K63-polyubiquitin chains. Yeast Rpn10Δ is viable and required for the degradation of specific substrates (e.g., Sic1) in conjunction with Rad23Δ [29]
Rpn10/S5a	Mammalian	Two UIM domains	Intrinsic 19S subunit, but can be substoichiometric	Binds K48 and K63-polyubiquitin chains [2]. Does not bind homotypic K11-ubiquitin chains [54]. Rpn10 deletion in mouse hepatocytes causes mild liver impairment [15]
Rpn13	Yeast/mammalian	Pleckstrin-like receptor of ubiquitin (PRU) domain	Intrinsic 19S subunit	Binds K48 and K63-polyubiquitin chains Rpn13 deletion in mouse hepatocytes causes mild liver impairment [15]. Severe liver disease and accumulation of ubiquitin conjugates is observed with deletion of both Rpn10 and Rpn13 [15]
Rpt5	Yeast/mammalian	Not known	Intrinsic 19S subunit	Reported to bind ubiquitin conjugates using crosslinking experiments [13], which may result from its close proximity to Rpn10 [74]
Dss1 (Sem1)	Yeast	Unstructured ubiquitin binding site (UBS)	Proteasome assembly	Binds polyubiquitin conjugates. Conserved in eukaryotes, but its ubiquitin binding function has only been examined in fission yeast [14]
Rad23	Yeast	Two UBA domains	Proteasome-associated. Binds to Rpn1	Combined Rad23Δ/Dsk2Δ causes accumulation of polyubiquitin conjugates and reporter substrates in yeast [75]
hHR23A	Mammalian	Two UBA domains	Proteasome-associated. Binds to Rpn10/Rpn13	Binds selectively to K48-polyubiquitin conjugates [21]
hHR23B	Mammalian	Two UBA domains	Proteasome-associated. Binds to Rpn10/Rpn13	Binds selectively to K48-polyubiquitin conjugates [21]. hHR23A and B have different substrates in mammalian cells [19]
Dsk2	Yeast	UBA domain	Proteasome-associated. Binds to Rpn10/Rpn13	Combined Rad23Δ/Dsk2Δ causes accumulation of polyubiquitin conjugates and reporter substrates in yeast [75]
UBQLN1 and 2	Mammalian	UBA domain	Proteasome-associated. Binds to Rpn10/Rpn13	Mutations in UBQLN are associated with MND [26]
Ddi1	Yeast/mammalian	UBA domain	Proteasome-associated. Binds to Rpn10/Rpn13	Ubl–UBA protein that may function similarly to Rad23 [23]

In addition to the intrinsic 19S ubiquitin receptors, the proteasome can bind polyubiquitinated substrates through proteins that transiently associate with the 19S (Fig. 1b; Table 1). Such proteins typically encode both ubiquitin like (Ubl) domains and UBDs. The UBDs bind the polyubiquitin chains, while the Ubl binds to the 19S high affinity ubiquitin receptors or other 19S subunits (e.g., Rpn1) [16]. Yeast Rad23 was one of the first Ubl–UBD proteins shown to bind polyubiquitinated proteins through its two UBA (*ub*iquitin-*a*ssociated) domains and deliver these conjugates to the proteasome for degradation [16, 17]. However, the relative importance of Rad23 in recruiting polyubiquitinated proteins to the proteasome has subsequently been questioned, as although initial studies suggested that Rad23 and its human homologues (hHR23A and B) bind to polyubiquitinated proteins and the proteasome [18, 19], others found that Rad23 prevented protein degradation [20]. It is likely that these differences in Rad23 function are due to the protein concentration used in the in vitro assays of proteasomal degradation. We found that when hHR23A and B are used at concentrations similar to those found in vivo, they stimulate the binding of K48-polyubiquitinated proteins to mammalian proteasomes [21].

Several other Ubl–UBA proteins have been identified, including Ddd1 [22, 23], Dsk2 [16, 24], and its human homologues [hPLIC1 and 2, also known as Ubiquilin (UBQLN1 and 2)] [25] (Table 1). The importance of these proteins is highlighted by the identification of human mutations causing neurological disease. Mutations in UBQLN2 cause an X-linked form of motor neurone disease [MND, or amyotrophic lateral sclerosis (ALS)] [26], while mutations in UBQLN1 are associated with MND as well as Alzheimer’s disease [27, 28]. These mutations are thought to be pathogenic in part due to the accumulation of ubiquitinated protein aggregates and defects in proteasomal degradation [26].

With the relatively large number of known ubiquitin receptors that are either encoded within the 19S or can associate with the complex (Table 1), it is not surprising that the deletions of individual UBPs may not alter viability [29, 30]. However, it is likely that different proteasome-associated UBPs bind distinct groups of polyubiquitinated proteins, allowing for selective and specific protein degradation. Indeed, Verma et al. show that while yeast deletion of Rad23 and Rpn10 does not affect overall protein degradation, it does prevent the degradation of specific proteasome substrates, such as Sic1 [29]. Similarly, in a mammalian system, Hamazaki et al. show that liver-specific deletion of either Rpn10 or Rpn13 results in modest impairment of function, but the deletion of both ubiquitin receptors causes severe liver injury and the accumulation of polyubiquitin conjugates [15].

## K48-polyubiquitin chains signal proteasome-mediated degradation

K48-polyubiquitin chains are the most abundant linkage in cells [31, 32], and are thought to be the major signal for proteasome-mediated degradation [33, 34]. Quantitative mass spectrometry analyses of intracellular ubiquitin linkages support this notion, as K48-polyubiquitin linkages rapidly accumulate when cells are treated with the proteasome inhibitor MG132 [32]. Biochemical studies provided the first direct evidence for K48-polyubiquitin chains binding to the 26S proteasome and stimulating protein degradation [33, 35]. Subsequently, Thrower et al. identified a chain of four or more K48-linked ubiquitins as the optimal chain length to stimulate protein degradation [35]. However, recent studies suggest that the number of K48-polyubiquitin chains may be more important than the chain length. In elegant proteasomal degradation assays and single molecule kinetic studies, Lu et al. demonstrate that multiple ubiquitinated lysines are more efficient than K48-polyubiquitin chains in stimulating proteasomal degradation [36]. They observed rapid degradation of the cell-cycle protein, cyclin B1, when modified with two diubiquitin K48-chains as compared with a single tetraubiquitin K48-chain [36]. It should be noted that human proteasomes used in these assays were salt washed to remove the DUB USP14 and associated proteins (including hHR23A and B). Whether Ubl–UBA proteins affect the degradation of ubiquitinated proteins modified with several K48-linked chains remains to be determined.

**Fig. 2:**
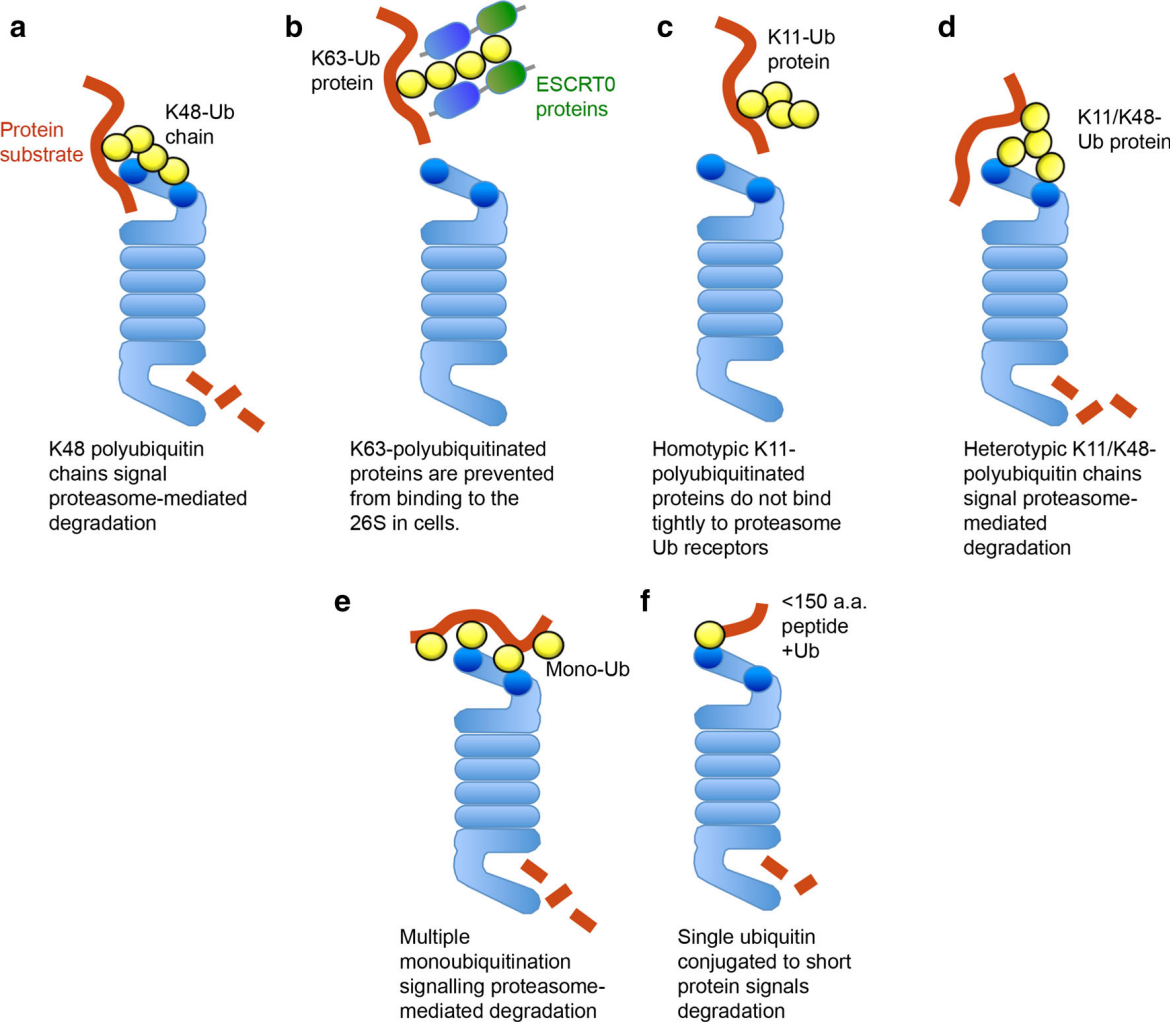
Recognition of different ubiquitin linkages by the 26S proteasome. Schematic of the interaction of the 26S proteasome with different types of ubiquitin linkages. The positions of Rpn13 and Rpn10 within the proteasome are highlighted (*dark blue*). K48-polyubiquitinated proteins are the canonical signal for proteasome-mediated degradation (**a**). K63-polyubiquitin chains are blocked from binding to the 26S due to K63-selective ubiquitin binding proteins, which bind tightly to the K63-polyubiquitin chains and direct them to alternative pathways (endosomal–lysosomal pathway) (**b**). Homotypic K11-polyubiquitin chains do not bind tightly to the 19S proteasome receptors (**c**). Heterotypic K11/K48-polyubiquitinated proteins signal proteasome-mediated degradation of cell-cycle substrates (e.g., cyclin B1) (**d**). Multiple monoubiquitination can facilitate the degradation of some proteasome substrates (cyclin B1) (**e**). Short proteins (less than 150 amino acids) may be targeted for degradation by a single ubiquitin moiety (**f**). *Ub ubiquitin*

Biochemical assays confirm that K48-polyubiquitin conjugates initially bind to the proteasome through Rpn10 and Rpn13 (Fig. 2a). Proteasomes purified from yeast strains that lack UBDs in Rpn10 and Rpn13 (rpn10∆UIM, rpn13KKD, or rpn10∆UIM/rpn13KKD) show reduced affinity for K48-polyubiquitin conjugates, with a fourfold decrease in affinity for the double mutant [2]. However, some K48-polyubiquitin conjugates still bind to proteasomes in these Rpn10/Rpn13 mutants, indicating the presence of lower affinity ubiquitin receptors for polyubiquitin chains [2].

Because K48-polyubiquitin chains are the major signal for proteasome-mediated degradation, it was possible that the intrinsic proteasome ubiquitin receptors selectively bind K48-linked chains. This is clearly not the case, as pure proteasomes bind K48- and K63-linked chains similarly, through the same high affinity Rpn10 and Rpn13 ubiquitin receptors [2, 37, 38]. However, Ubl–UBA proteins, such as Rad23, do show selectivity for K48-polyubiquitin chains. The second UBA domain of Rad23 has a higher affinity for K48-tetraubiquitin compared with K63-tetraubiquitin [39]. In addition, we found that full-length hHR23A and hHR23B bind only to K48-polyubiquitin conjugates when compared with K63-polyubiquitin chains [21]. Furthermore, full-length hHR23A and, particularly, hHR23B selectively stimulate K48-polyubiquitin conjugates binding to the 26S proteasome [21]. Therefore, the Rad23 proteins do provide ubiquitin linkage specificity for delivering K48-polyubiquitinated proteins to the proteasome. Whether other proteasome-associated ubiquitin receptors share the same specificity for K48-linkages remains to be determined.

## K63-polyubiquitin chains bind to pure proteasomes but do not signal degradation in cells

K63-polyubiquitin linkages are also highly abundant in cells [31, 32], but serve alternative functions to proteasome-mediated degradation, and are involved in intracellular signalling [40], DNA repair [41], and the targeting of proteins to the endosomal–lysosomal system [42]. One could, therefore, hypothesise that K63-polyubiquitinated proteins cannot bind to the proteasome. However, as previously mentioned, K63-polyubiquitin conjugates do not only bind pure 26S proteasomes [2, 21] in vitro, but can also stimulate protein degradation at similar rates to K48-polyubiquitinated substrates [37, 38].

Three mechanisms could explain this discrepancy between the function of K63-polyubiquitinated chains in cells and their ability to signal proteasome-mediated degradation in vitro: (1) K63-polyubiquitin chains are rapidly disassembled in cells preventing their association with the 26S proteasome. (2) K48-polyubiquitin chains are more efficiently delivered to the proteasome by proteins, such as hHR23B, and (3) K63-polyubiquitin chains are blocked from binding the 19S by intracellular factors. Several groups have examined which of these mechanisms may account for preventing the degradation of K63-polyubiquitin conjugates in cells.

Cooper et al. found that K63-polyubiquitin conjugates in cell lysates were rapidly disassembled compared with K48-chains, and identified the BRISC (Brcc36 isopeptidase complex) as the DUB responsible for this observation [43]. Jacobson et al. also found that K63-polyubiquitin chains are more rapidly disassembled by the proteasome-associated DUBs [31]. The proteasome DUB Rpn11 (Poh1) and BRISC are members of the JAMM/MPN+ (Jab1/Mov34/Mpr1 Pad1 N-terminal+) proteases, and it has been suggested that this group of DUBs shows a preference for K63-polyubiquitin chains [43]. However, it is unlikely that rapid disassembly of K63-conjugates provides the major mechanism for protecting K63-polyubiquitinated proteins, as the binding of polyubiquitin chains to the high affinity proteasome ubiquitin receptors precedes DUB activation. Furthermore, by measuring conjugate binding at 4 °C, we showed that both K48- and K63-polyubiquitin conjugates bind similarly, and we observed no difference in this binding when DUB inhibitors were added [21].

To identify factors in cells that may prevent K63-polyubiquitin chains from binding to the proteasome, we used a biochemical and mass spectrometry approach. Several proteins that bound strongly to K63-polyubiquitin conjugates were identified, including the ESCRT0 proteins [21]. This heterodimeric complex recruits polyubiquitinated proteins to the endosomal–lysosomal pathway (reviewed in [44]), but is also present within the cytosol [21]. We found that ESCRT0 not only selectively bound to K63-polyubiquitin chains, but also prevented these conjugates from binding to pure proteasomes. Thus, K48-selective Ubl–UBA proteins (e.g., hHR23B) stimulate K48-polyubiquitin conjugates binding to the proteasome, and K63-polyubiquitin chains are protected from proteasomal degradation by K63-linkage-specific UBPs, such as ESCRT0 (Fig. 2b) [21].

## Homotypic K11-polyubiquitin chains do not bind to the proteasome

K11-linked polyubiquitin chains are the third most abundant linkages in cells [32], and have a unique structure compared with K48, K63, or linear-linked chains [45]. The most well characterized role for K11-linkages is in the cell cycle. In mitosis, the anaphase promoting complex/cyclosome (APC/C) E3 ligase catalyzes polyubiquitin chain formation on cell-cycle proteins (e.g., cyclin B1 and securin) signalling the degradation of these mitotic regulators [46, 47]. K11-chains have also been shown to accumulate in cells released from mitotic arrest, when the APC/C is most active [48]. In vitro studies have identified that several E2 enzymes are required for the ubiquitination of APC/C substrates. Either UbcH10 or UbcH5 is required to initiate ubiquitination of cyclin B1, and the polyubiquitin chain formed is then extended by the K11-specific E2 enzyme Ube2S [48, 49]. It was, therefore, initially thought that K11-chains were required for the degradation of cell-cycle checkpoint proteins. Interestingly though, knockdown of Ube2S by siRNA-depletion in HeLa cells only delayed exit from mitosis when drug-induced perturbations (e.g., monastrol) of the cell cycle were used, and Ube2S was not required for normal mitosis [49]. In addition, Ube2S is not necessary for degradation of ubiquitinated cyclin B1, as multiple monoubiquitination of cyclin B1 was sufficient to initiate its proteasome-mediated degradation [36, 50].

In contrast to the presumed proteasome-mediated degradative role of K11-linkages in the cell cycle, K11-polyubiquitination has been observed in other cellular processes, including ER-associated degradation (ERAD) [32], the hypoxia response [51], mitophagy [52], and even in stabilizing proteins, such as β-catenin [53].

Using pure 26S proteasomes and homotypic K11-polyubiquitin conjugates, we examined whether these chains facilitated proteasomal degradation. Surprisingly, we found that homotypic K11-chains did not bind to pure proteasomes or to the proteasome-associated ubiquitin receptors (Fig. 2c) [54]. In addition, homotypic K11-polyubiquitin chains did not facilitate the rapid degradation of cyclin B1. The finding that homotypic K11-polyubiquitination does not signal proteasomal degradation has been subsequently observed by Martinez-Fonts et al, who used a fluorescent-based proteasomal degradation assay of K11-polyubiquitinated GFP [55].

We also examined the binding of homotypic K11-polyubiquitin chains to the 19S ubiquitin receptors and Ubl–UBA proteins. Consistent with K11-polyubiquitin chains being a weak signal for proteasomal degradation, we observed no significant binding of homotypic K11-chains to Rpn10, hHR23A, hHR23B, or UBQLN1 [54]. Other groups have examined the direct binding of UBDs within proteasome-associated ubiquitin receptors and K11-chains. Castaneda et al. show that the UBA2 domain of hHR23A binds K48 dimers more strongly than K11-ubiquitin dimers [45]. It appears that a single molecule of UBA cannot interact with both hydrophobic regions of the K11-ubiquitin dimers at the same time. This is not the case for K48 ubiquitin dimers, which can associate with a single UBA moiety [45]. The compact K11-ubiquitin dimer formation and orientation of the two ubiquitin molecules may prevent the I44 hydrophobic region being available to the proteasomal ubiquitin receptors, hence explaining the weak binding of these chains in comparison with K48-ubiquitin linkages [54].

## Heterotypic K11/K48-polyubiquitin chains signal proteasome-mediated degradation

It is likely that the diverse fates of K11-polyubiquitinated proteins relates to the homotypic or heterotypic conformation of the chain. Recent studies show that the APC/C forms heterotypic K11/K48-chains on cell-cycle substrates (Nek2A and cyclin A), rather than homotypic K11-chains [56]. Mayer et al. observed that these heterotypic chains were more efficient at stimulating the degradation of the cell-cycle substrates compared with homotypic K48-polyubiquitin chains (Fig. 2d) [56]. While we also found that heterotypic K11/K48-polyubiquitin chains bind to the proteasome and signal degradation, they were not as efficient as homotypic K48-polyubiquitin chains [54]. The reason for this discrepancy may relate to the experimental conditions and our use of recombinant APC/C rather than APC/C immunoprecipitated from cell extracts.

The ability of the proteasome to differentially degrade homotypic and heterotypic K11-polyubiquitin conjugates may also relate to disassembly of the chains by the proteasome-associated DUBs. We observed that K11/K48-heterotypic chains were disassembled slowly compared with the K11-homotypic chains [54]. Mansour et al. also found that K11-polyubiquitin chains were rapidly disassembled by the proteasome-associated DUBs compared with K48-chains. How the heterotypic chains bind and interact with the proteasome-associated DUBs is unclear. Furthermore, whether K11-heterotypic chains containing other lysine linkages can be disassembled by these DUBs, or stimulate proteasomal degradation is not known. However, it is noteworthy that complex polyubiquitin chains, containing forked chains of mixed linkages have been reported to block proteasomal degradation [57].

## Monoubiquitination as a signal for proteasome-mediated degradation

Monoubiquitination is typically involved in subcellular localization of proteins [58] or signalling the internalization of plasma membrane receptors, rather than facilitating degradation [59]. However, the requirement for a polyubiquitin chain of four or more ubiquitin moieties has been challenged by several recent studies. Dimova et al. showed that multiple monoubiquitination of cyclin B1 is an efficient signal for proteasomal degradation in *Xenopus* extracts (Fig. 2e) [50]. Lu et al. confirm that multiple monoubiquitination of cyclin B1 is sufficient to stimulate degradation by human proteasomes using single molecule kinetic studies [36]. However, multiple monoubiquitination of other cell-cycle substrates (geminin and securin) did not facilitate rapid proteasomal degradation [36]. The distribution and conformation of the multi-monoubiquitinated protein may be important for binding to the proteasome ubiquitin receptors. In addition, the position of unfolded regions within the ubiquitinated substrate will contribute to the rate of proteasomal degradation [60]. Indeed, Shabek et al. show that a single ubiquitin fused to an approximately 150 amino acid polypeptide (repeat HA tag) was sufficient to signal proteasomal degradation (Fig. 2f) [61]. However, ubiquitin fused to polypeptides longer than 150 amino acids were degraded slowly.

## What is the role of other ubiquitin linkages in signalling proteasomal degradation?

Whether the other ubiquitin linkages that occur in cells can mediate proteasomal degradation is largely unknown. Linear chains are structurally similar to K63-polyubiquitin chains, and appear confined to NF-κB signalling [62]. UBPs, such as NEMO (NF-κB essential modulator), selectively bind linear chains [63], and may prevent them binding to the proteasome, similarly to ESCRT0 and K63-polyubiquitin chains [21].

While K6, K27, K29, and K33 ubiquitin linkages are all detected at low levels in cells, they do all increase to varying extents when cells are treated with proteasome inhibitors [32]. This suggests that they may be involved in proteasome-mediated degradation, but not necessarily in homotypic conformations. However, the role of these ubiquitin linkages in cells remains unclear. K6 and K27 linkages are associated with DNA replication repair [64, 65], while K29 and K33 are hydrolyzed by the DUB involved in Wnt signalling (TRABID) [66]. Structural analyses of dimeric forms of these atypical ubiquitin linkages reveal distinct conformations [67–69], suggesting that they can form unique ubiquitin signals. Whether they have a role in proteasome-mediated degradation remains to be determined.

## Future perspectives

Advances in biochemical techniques and single molecule kinetics will offer further mechanistic insights into ubiquitin recognition by the 26S proteasome. Particularly, the ability to form different polyubiquitin linked chains of defined length will help to elucidate the role of atypical ubiquitin linkages in proteasome-mediated degradation. However, determining the type and number of ubiquitin chains that occur on individual protein substrates in cells remains a challenge. Post-translational modifications of ubiquitin and its receptors add a further level of complexity to the system. For example, phosphorylation of ubiquitin at serine 65 (S65) by PTEN-induced putative kinase 1 (PINK1) is required for the clearance of damaged mitochondria [70–72]. While S65 phosphorylated ubiquitin can be incorporated into different polyubiquitin chains [73], the role of the proteasome in degrading such conjugates is not known. It will be of interest to examine how S65 phosphorylation and other modifications of ubiquitin are involved in proteasome-mediated degradation.
